# ARCCH Model of Resilience: A Flexible Multisystemic Resilience Framework

**DOI:** 10.3390/ijerph19073920

**Published:** 2022-03-25

**Authors:** Armeda Stevenson Wojciak, Jan Powers, Athena Chung Yin Chan, Allison L. Pleggenkuhle, Lisa M. Hooper

**Affiliations:** 1 Department of Family Social Science, College of Education and Human Development, University of Minnesota, St. Paul, MN 55107, USA; chan1850@umn.edu; 2 Department of Education, Creighton University, Omaha, NE 68178, USA; janpowers@creighton.edu; 3 Center for Educational Transformation, University of Northern Iowa, Cedar Falls, IA 50614, USA; pleggena@uni.edu (A.L.P.); lisa.hooper@uni.edu (L.M.H.)

**Keywords:** resilience, framework, culturally informed, culturally-responsive, systemic, trauma, adverse childhood experiences, ARCCH Model of Resilience

## Abstract

The increasing prevalence and impact of trauma, such as adverse childhood experiences, race-based trauma, and a global pandemic, highlight the critical need for a flexible multisystemic framework of resilience. This manuscript outlines the universality of trauma and resilience and also provides a description of the gaps in existing resilience frameworks that led to the development of a flexible multisystemic resilience framework entitled the ARCCH Model of Resilience. Attachment, Regulation, Competence, Culture, and Health are elements of personal and cultural identities, families, communities, and systems that can be used to evaluate strengths, identify areas that need support, and provide steps for culturally responsive and ecologically valid interventions. A multisystemic application of ARCCH is provided.

## 1. Introduction

Among national samples of Americans, scholars have consistently demonstrated that experiencing trauma is relatively common with most people experiencing at least one traumatic event [1]. While some types of traumas, such as natural disasters, may not be avoidable, most interpersonal violence, adverse childhood experiences (ACEs), and race-related traumas can be prevented, and the effects mitigated. In this paper, we propose a flexible multisystemic resilience model entitled the ARCCH Model of Resilience. ARCCH stands for attachment, regulation, competence, culture, and health. Within the ARCCH Model of Resilience, we acknowledge the prevalence of trauma that any individual may experience and how that individual brings their experiences and cultural identities into all their interactions within their relationships. We suggest using the ARCCH Model of Resilience to support resilience of individuals and the systems they inhabit holistically. In this paper, we will briefly review the universality of trauma, the importance of resilience, the ARC Framework, and the much-needed adaptations of the original model to include health, culture, a broader system understanding, and an application of the model. The resulting ARCCH Model of Resilience is a comprehensive, culturally informed, culturally responsive, and holistic framework that aims to mitigate the effects of diverse types of trauma evinced in individuals and systems and build resilience among individuals and systems.

### 1.1. Trauma

According to Substance Abuse and Mental Health Services Administration, trauma is defined as an event, series of events, or set of circumstances that is experienced by an individual as physically or emotionally harmful or life threatening and that has lasting adverse effects on the individual’s functioning and mental, physical, social, emotional, or spiritual well-being ([2], p. 7). Substantial research has demonstrated that trauma has a long-term impact over individuals’ physical and psychological well-being [3,4]. It is evident that ACEs are disproportionately more frequent and prevalent in communities of color [5,6]. Within the United States, 61% of Black children and 51% of Hispanic children experienced at least one ACE, compared with 40% of White children and only 23% of Asian children [6]. However, trauma goes beyond individual experiences to family, community, and multisystem experiences, such as domestic violence, race-related trauma, and the global coronavirus disease 2019 (COVID-19) pandemic. Thus, we challenge readers to conceptualize trauma from a broader multisystemic perspective.

An ecological framework enables a conceptualization of how trauma is implicated in both individuals and systems [7]. Individuals may experience trauma from the complex interplay between close relationships, community, and society. Individuals do not act in isolation but interact with different close relationships (e.g., family, peers), communities (e.g., school, workplaces, and neighborhood), and society broadly (e.g., cultural norms). Traumas and other ways of conceptualizing trauma such as ACEs penetrate different layers of the social-ecological model, from close relationships (e.g., abuse and neglect, domestic violence), community (e.g., bullying, community violence), to society (e.g., ethnic/racial discrimination, refugees and forced migration due to wars or conflicts). In fact, racism or racial trauma could be carried to the next generation because of genetic predisposition and socioenvironmental factors, as well as the underlying cultural norms [2], thus, highlighting the need for a culturally informed ACEs model. Bernard and colleagues proposed C-ACE (culturally informed adverse childhood experiences) model to illustrate the pervasive and deleterious mental health impact of racism on racial and ethnic minority populations [8]. Given that we may not have knowledge of the trauma history of children and affiliated others, emerging research calls attention to a comprehensive framework to promote multisystemic resilience [9].

### 1.2. Resilience

There are three main types of resilience: individual resilience, family resilience, and community resilience. Baker, Baker, and Burrell [10] described individual resilience, or personal resilience, as a process by which an individual utilizes their strengths and mental processes to adapt to or overcome stressful situations or adversity. Maurović, Liebenberg, and Ferić defined family resilience as a process consisting of a risk initiating the resilience process, protective factors facilitating the resilience process, and good outcome(s) despite risk exposure within a family system [11]. A family in this context is considered to be any system by which at least two or more people interact in a way they define as familial. Ellis and Dietz explained community resilience as a process by which a community can anticipate risk, limit effects, and rapidly recover through adaptation to community-wide trauma and adversity [12]. The Milken Institute School of Public Health conceptualized the Building Community Resilience (BCR) Model to develop community resilience by “creating a shared understanding of childhood and community adversity, assessing system readiness, developing cross-sector partnerships, and engaging families and residents” ([13], p. 3).

It is important to consider all three types of resilience as individuals exist within many different systems and will likely experience individual, family, and community trauma within their lifetime; furthermore, it is important to consider how to best build resilience in all systems. In fact, broadening our understanding of resilience is paramount to a more holistic conceptualization of how to best support resilience, especially in a culturally responsive and trauma informed way. Masten and colleagues proffered a systemic lens and defined resilience as “the capacity of a dynamic system to adapt successfully through multisystem processes to challenges that threaten the function, survival, or development of the system” ([14], p. 524). Fitzgerald and colleagues discuss the importance of several multisystemic processes that are important to racially and ethnically diverse individuals, families, and communities [15]. The processes they reviewed that help promote resilience were the importance of family and community networks, racial socialization, cultural embeddedness, spirituality, sense of belonging, and the importance of elders or those that help to maintain cultural traditions, and forms of resistance for promoting resilience. Resistance is seen as a way of returning social agency to racial and ethnic communities. Through this dynamic process are opportunities to support and strengthen individuals and systems to build resilience. The Attachment, Regulation, and Competence (ARC) Framework is a flexible intervention designed to support children and adolescence who have experienced trauma [16,17].

### 1.3. Attachment, Regulation, and Competence Framework

The Attachment, Regulation, and Competence (ARC) Framework was originally developed as an intervention to address the complicated needs of children and adolescents’ who have experienced trauma by working with the clinician and the youth’s caregiver [16,17,18]. The ARC Framework has also been used across service settings to help systems change to support the youth [18]. The ARC Framework is theoretically grounded in four areas of science: normative childhood development, attachment, traumatic stress, and risk and resilience. The ARC Framework identifies three core domains: attachment, regulation, and competence that are impacted by trauma and that can be supported through intervention [16,17,18]. The ARC Framework was originally designed for children and adolescents; however, over time it has been used with individuals from birth to young adulthood and across different levels of cognitive functioning.

The ARC Framework was first applied in statewide clinical settings for children and youth impacted by trauma [16,17,18], child welfare or juvenile justice residential settings [19,20,21,22], and education settings [23]. Most investigations targeted the mental health (i.e., trauma symptoms such as depression, anxiety, post-traumatic stress disorder) of predominantly White children and adolescents. Scholars have indicated the effectiveness of the ARC Framework in reducing mental health in a cohort of younger children involved with the child welfare system with diverse ethnocultural backgrounds. The authors state that as part of trauma treatment it is important to be attuned to systemic and cultural factors as well as therapeutic context. The authors stressed the importance of discussing cultural differences and highlighted the negative impact cultural misattunement could have between a youth and caregiver [24]. The evidence supporting the ARC Framework has demonstrated the usefulness of this intervention in supporting children and adolescents who have experienced complex trauma, their caregivers, and the systems that support them [16,17,18]. However, the overall application of the ARC Framework lacks the explicit consideration and integration of diverse ethnocultural backgrounds of youth, health development, and the systems they inhabit. In response to these limitations, we have posed an adapted resilience model that is culturally responsive, trauma-informed, and can be applied to an individual, family, community, and/or system. Below is our rationale for the addition of culture and health to create the ARCCH Model of Resilience.

### 1.4. Importance of Addressing Culture in a Resilience Framework

The implications of culture—broadly defined—on diverse traumatic experiences, resilience, and other processes and outcomes has never been more urgent. The recent COVID-19 pandemic has exposed—once again—how racial and cultural identities and cultural contexts (risk prone ecological contexts) are implicated in differential treatment experienced by racial, ethnic, and language minorities among other populations [6,8]. Given that ACEs and other traumatic experiences disproportionately impact racial minority populations, this lack of attention to culture is a significant gap in the empirical, theoretical, and clinical literature; in turn, there is also a gap in what is known about what works and the number of available empirically supported, culturally responsive, and trauma-informed models [25,26]. What is established in the accumulated literature—composed of primarily White American samples—is how attachment, regulation, and competence are factors that can be affected by trauma and how they can be promotive factors for resilience. We contend these factors, both separately and jointly, cannot be fully understood without the consideration of culture.

#### 1.4.1. Cultural Identities

In the newly released guidelines, the American Psychological Association [27] defines culture as “the values, beliefs, language, rituals, traditions, and other behaviors that are passed from one generation to another within any social group. Broad definitions include any socially definable group with its own set of values, behaviors, and beliefs. Accordingly, cultural groups could include groups based on shared identities such as ethnicity (e.g., German American, Blackfoot, Algerian American), gender (e.g., women, men, transgender, gender-nonconforming), sexual orientation (e.g., gay, lesbian, bisexual), and socioeconomic class” (p. 11). As we consider the cultural identities commonly discussed in the literature (e.g., age and generation, disability, ethnicity/race, sexual orientation, socioeconomic status, language, and gender; [28]), we are compelled to underscore that the “problem” is not a specific cultural identity (e.g., race, gender, GLBTQQI) or the intersection of those identities but the toxic stress and structural and systemic issues many racial, ethnic, and cultural minority individuals experience that is the problem and implicated in a range of outcomes (e.g., mental and physical health). To be clear, individual cultural identities (age, gender, race) are important factors in how resilience frameworks may be culturally tailored.

#### 1.4.2. Cultural Context

Scrine [29] and others [26,30] highlight the importance of the cultural context (ecological, sociopolitical, organizational). In most cases, a consideration of cultural context is important because many current systems and contexts in the United States can be both trauma producing and trauma reducing [26,30]. Being trauma-informed requires a consideration of how the system and organization in which the model is being employed (e.g., ARCCH Model of Resilience) may be trauma producing (see [28,31]).

Using the K-12 school system and culture as an example, it would be critical to consider how the school system may simultaneously mitigate further trauma and promote further resilience for students, school personnel, and the school practices, policies, and procedures. A lack of consideration for how the K-12 school system (and other systems) have been implicated in trauma would be a significant misstep in the case of any trauma-informed approach (see [31]). The same argument about the importance of any organizational ecology (K-12 school system) could be proffered about the sociopolitical and geographical ecologies (see [28]). Henfield and colleagues contended that “It feels incomprehensible to divorce operational definitions of trauma from the current sociopolitical landscape” (p. 1). Thus, efforts toward ameliorating trauma and establishing trauma-informed care models must consider racism—in all its forms—and other means and acts of intersecting oppressions that have long existed in social and organizational systems and cultural contexts [28].

Henfield and colleagues have outlined how the cultural context(s) and the cultural identities of individuals and communities intersect and are implicated in both trauma and the additive and aftereffects of trauma [28]. Additionally, researchers, practitioners, educators, and policy makers must be aware that the pernicious additive, intersectional effects of trauma are disproportionately evidenced in racial, ethnic, cultural, and linguistic minority populations as compared to White American populations. Thus, all trauma-informed care models will lack efficacy, effectiveness, and in the end, will fail if culture is not a seminal aspect of the model. Culture must be considered at individual (e.g., GLBQTIA people), organizational (e.g., primary care systems), and systemic (e.g., practice, policies, and procedures) levels.

### 1.5. Importance of Addressing Health in a Resilience Framework

Just as attachment, regulation, and competence are factors that can be affected by trauma but can also be promotive factors for resilience, so is the construct of health. The trauma-informed movement was largely sparked by the ACE study, which demonstrated a significant relationship between childhood trauma and long-term negative health effects throughout adulthood. This original ACE research investigated the relationship of health risk behavior and disease in adulthood to the extent of exposure to childhood trauma. Several categories of adversity were studied including psychological, physical, or sexual abuse; violence against the mother; or household dysfunction, such as, living with someone with problems related to substance abuse, mental illness, or incarceration. The number of these categories that were experienced were compared to measures of adult risk behaviors and health status, using logistic regression to adjust for effects of demographic factors. The results showed that ACEs are very common; the higher the number of categories experienced, the higher the likelihood of developing long-term health problems, such as heart disease, cancer, and diabetes, which are some of the leading causes of early death [1].

The ACE study continues as an ongoing collaboration between Kaiser Permanente and the Centers for Disease Control and Prevention [32], demonstrating a correlation between early adversity and health. Many replications continue to find this strong relationship between trauma and multiple risk factors for serious health issues across the life span [33,34]. In addition, the number of adults reporting childhood adversity is increasing, leading to an increased focus on prevention [35] and building multisystemic resilience [36].

As evidence continues to mount on the lifelong impact trauma has on health, other study areas of trauma and resilience are being funded to investigate this connection [33,35]. Scholars have demonstrated how the effects are particularly deleterious when adversity was prolonged, such as with toxic stress, or occurring during sensitive times of neurobiological development [1,30,37,38,39]. In addition, researchers have studied protective factors of resilience that mitigate the negative effects on health, finding that skills in emotional regulation and connectedness to others were strong protective factors of resilience that led to better health outcomes [37]. Therefore, a useful model of resilience must consider the construct of health both as a trauma symptom requiring support and/or a possible resilience strength.

## 2. The ARCCH Model of Resilience: An Expanded Conceptual Framework

As depicted above, the importance of explicitly incorporating culture and health into a resilience framework is imperative and currently lacking. Consequently, we built from the ARC Framework [16,17,18] to develop the ARCCH Model of Resilience, a flexible multisystemic resilience framework to help individuals, families, communities, and systems promote resilience through this lens. The ARCCH Model of Resilience consists of attachment, regulation, competence, culture, and health. Below is our conceptual model, followed by definitions we ascribe to each construct, the assumptions that inform our thinking and applications, and lastly an example of how the ARCCH Model of Resilience can be applied in different contexts.

### 2.1. The ARCCH Model of Resilience—Conceptual Model

**Figure 1:**
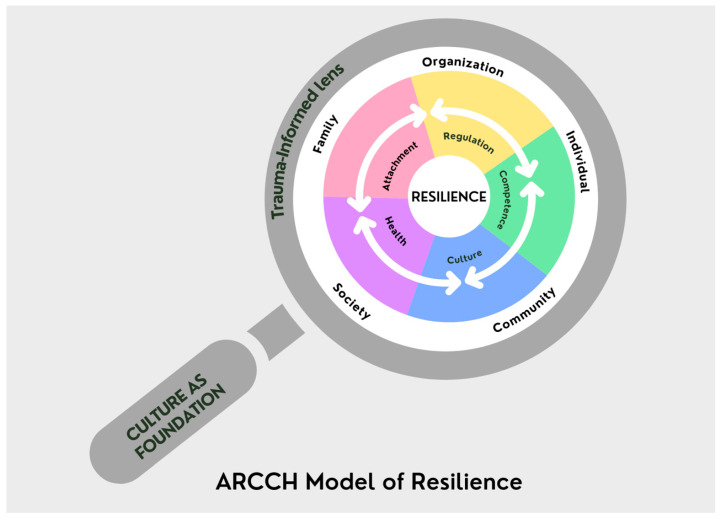
The ARCCH Model of Resilience: A Flexible Multisystemic Resilience Framework. In this figure, we illustrate how The ARCCH Model of Resilience can be an important tool to support resilience. This tool is depicted as a magnifying glass to signify that the ARCCH Model of Resilience is the lens that informs their way of being and seeing how to support resilience in individuals, families, communities, and systems. The handle of the magnifying glass signifies that culture (cultural identities and cultural context) is a necessary component to use the tool. Without this consideration you will not be able to appropriately use the tool/framework. Then, notice that a trauma-informed lens holds the interconnected components of the ARCCH Model of Resilience. You cannot truly understand the attachment, regulation, competence, culture, or health of an individual, family, community, or system without considering the culture and trauma that influence the systems they inhabit. Once these considerations are understood, then one can begin to explore each ARCCH construct individually and the interconnection of the ARCCH components (attachment, regulation, competence, culture, and health) with those you are supporting to make up a plan to build the resilience of those you are supporting with the ARCCH Model of Resilience.

The ARCCH Model of Resilience is designed to be a strengths-based flexible model that can be used to build resilience of individuals, families, communities, and systems. In Figure 1, we provide an image to help explain the use of this conceptual model by someone who is supporting the individual, family, community, or system. It is the lens/framework they will use to help ensure that their way of being is true to the model. The application of the ARCCH Model of Resilience is dependent upon who/what system is the focus of needed support. Depending upon your focus—the individual, family, community, or system, you use the ARCCH Model of Resilience to navigate different pathways to promote resilience. Explicitly, we believe that the individual, family, community, and system have their own ARCCH constructs that can be explored and supported to promote resilience. The ARCCH Model of Resilience and the different pathways to promote resilience are undergirded by culture (the foundation of Figure 1 and incorporated into the five ARCCH components at the center of the magnifying glass), situated within a systems framework (the first circle of concentric circles), and a trauma-informed lens (the outer layer of concentric circles).

### 2.2. Definitions Underlying the ARCCH Model of Resilience

Below is an overview of the way in which we define and conceptualize the core components of the ARCCH Model of Resilience as well as the ways in which we understand systems. These constructs have implications for and transportability to both individuals (children, adolescents, and adults) and systems.

#### 2.2.1. Attachment

Attachment can be disrupted at any stage of development and because we know that having at least one trusted adult who is there for the child is associated with resilience [14], we wanted to broaden our understanding of who (trusted adults) and what (other connections) we are thinking about when conceptualizing attachment and what it means when supporting individuals and systems. Beyond the caregiver/child relationship, researchers have studied how attachment is related to connection with others beyond the family and in the environmental context, such as the teacher-child relationship and school attachment (connectedness and feeling of belonging demonstrated through positive relationships with peers and teachers; [40,41,42]).

In the ARCCH Model of Resilience, our conceptualization of attachment continues to use the view as described above but to also includes what helps people feel attached in a community, such as, having a sense of belonging or feeling a connection to the mission or vision. This broadened conceptualization is also helpful for addressing attachment issues in individuals whose trauma has negatively affected their ability to attach in healthy relationships with people [41,43]. For example, employers can still strengthen attachment with employees through building a sense of belonging at their organization through community rituals or celebrations and increased positive connection. The sense of belonging is important for families as well. In a study of playgroups for children, parents talked about the importance of belonging and being in a playgroup that supports their identity or culture and where they feel safe and included [44]. Escalera-Rayes [45] advocates for the importance of assessing and considering one’s feelings of attachment and sense of belonging within the group to understand the socio-ecological system. Together, we suggest that attachment within and to the system is an important consideration. For the ARCCH Model of Resilience, expanding the role of attachment through other adults and systems is important for building systemic resilience [23,46,47]. Please note that in this broadened conceptualization, we noted that attachment is not limited to parent-child, but something we think about for all involved in the system.

#### 2.2.2. Regulation

In addition to emotion, regulation also refers to the regulation of brain processes, such as executive functions skills. According to Zelazo and colleagues, executive functioning is “attention-regulation skills that make it possible to sustain attention, keep goals and information in mind, refrain from responding immediately, resist distraction, tolerate frustration, consider the consequences of different behaviors, reflect on past experiences, and plan for the future” (p. 1). As researchers have shown executive function skills to be malleable and capable of being strengthened or weakened by positive or negative experiences [48], they are a key component of regulation in the ARCCH Model of Resilience for mitigating trauma and building resilience. Self-regulation as a problem area for people who experienced trauma is supported in the combined work of epidemiology and neurobiology and can be seen in abnormalities in structural brain imaging [49,50]. When caregivers in families or those in schools, healthcare, communities, organizations, and systems are provided with information to understand challenging behaviors as possibly disrupted attachments or disrupted regulation due to the neurobiology of trauma [51], a shift in attitudes away from an authoritarian or punitive approach may help break the cycle of escalation [46]. For example, when one understands the flight-fight-freeze response during which dysregulated people have impaired access to the prefrontal cortex for problem solving, this may prevent triggering the adult into a dysregulated response and instead provide a calming response [37,46,51]. Then, with that understanding one may respond by acknowledging that the person has been activated and then consider how to support the individual while not escalating their response.

Further, as the ARCCH Model of Resilience is a flexible model across domains and not only for the individual, regulation also applies to systems in addition to the internal regulation of the brain in individuals. For example, just as the brain regulates the actions of the individual, groups can perform regulations to control actions in a community. The policies and procedures of any community, whether a classroom, a school, a human service agency, or the whole community, provide structure to maintain safety, consistency, and balance the freedom and rights of its members to achieve common goals. For example, Blitz, Yull, and Clauhs [52] used the Sanctuary Model [53] to assess school culture as a foundation for trauma-informed approaches in urban schools. Ford and Blaustein [19] used systemic regulation in a residential setting for juvenile justice targeting the entire system for building regulation.

#### 2.2.3. Competence

In the ARCCH Model of Resilience, outside of the clinical context, competence can refer to any assets or promotive factors of resilience, including skills, successes, and strengths [54]; as well as things beyond just social competence. Competence is also useful for assessing what supports are needed in the setting one is in. When individuals build their competence, it also builds their self-efficacy and provides a catalyst for continued growth and well-being [42]. A common belief is that when people have ability but are not performing up to their expected level of competence, it is due to a lack of motivation. However, Howse and colleagues [55] found that motivation alone was of limited value in boosting achievement for students unless accompanied by self-regulation, or a person’s ability to control the quality and sequence of their behaviors in task settings. Examples of self-regulation skills that boost competence are behaving carefully and reflectively, planning independently, maintaining focus, and handling strong feelings [48]. In the school setting, if students can exhibit a regulation of their emotions and behaviors in the classroom, not only does their academic competence increase, but teachers view them more positively and expect higher academic success, contributing to increased attachment and competence [46,53,55].

Not only does the ability to self-regulate improve academic outcomes for students, but this also applies to adults as well. Taxer and Gross [56] researched the relationship between teacher responses in self-regulation and the effect on their practice. They found that their ability to modulate their emotions to achieve various goals in their work was most often employed through the modulation strategy of suppression, both for themselves and for modulation of the emotions of students in their classroom. Taxer and Gross postulated that teachers employed this strategy to maintain a more conducive learning environment. According to van der Heijde [57], with the rise in online learning and employment through technological advances, self-regulation has been increasingly equated with the term employability and serves as a bridge between multiple employability theories. Self-regulation, according to van der Heijde, is a key component of career resilience as it is equated with the use of strategies, goal setting, emotional control, and social competence, resulting in favorable career outcomes. In the workforce, adults with high ACE scores were at much higher risk of problems with regulation that result in poor work performance and increased absenteeism [50], concepts often associated with incompetence.

#### 2.2.4. Culture

First, the explication of culture in the ARCCH Model of Resilience is two-fold: (a) culture is a part of the ARCCH Model of Resilience and (b) culture underpins the application of the model. Specifically, in our ARCCH Model of Resilience, we consider culture in two ways: both at the individual level and more broadly at the system and contextual level. We believe both are equally important in establishing the most inclusive, racially, culturally, and clinically responsive framework and ensuring the framework has relevance for addressing trauma and promoting resilience across individuals and systems. By adding culture to the ARCCH Model of Resilience (see Figure 1), we consider historical trauma in conjunction with ACEs and other traumatic experiences. Similarly, to ACEs and other traumatic experiences, the empirical literature shows that historical trauma is related to a range of deleterious health outcomes and disparities. The aftereffects of historical trauma can be transmitted from generation to generation and the additive effects of cultural identities have been discussed in the literature. The ARCCH Model of Resilience offers a conceptual and clinical culturally responsive framework that can fill a gap and help individuals, organizations, and systems. Without first considering culture and the different ways in which cultural identity and context (e.g., race, socio-economic status, geographic location, beliefs and practices, language) intersects within each person, family, community, and system, we would not have a complete understanding of or all of the information required to fully support the individual, family, community, or system’s resilience. In the ARCCH Model of Resilience, it is imperative for the person using the model to be culturally sensitive and able to broach culture, race, and ethnicity [58,59,60] with whomever is the focus of the support to understand the various ways in which culture (e.g., currently or historically) may be influencing the individual, family, community, or system. The attention to and inclusion of culture is essential to the way of being of the person facilitating the conversation and supports. Further, the attention to cultural identities and cultural context are incredibly important as scholars are revisioning what resilience means and how it appears among racially and ethnically diverse families, particularly Black individuals and families in the United States [60]. Within the revisioning process is the need for culturally responsive ways of being when working with individuals and families [60].

In the ARCCH Model of Resilience, we recognize the numerous experiences (e.g., neglect, emotional abuse, observing interpersonal violence, parental substance abuse) that can be characterized as traumatic and at the same time—until the recent past—often went underrecognized and uncategorized as an adverse child experience and/or a traumatic experience rather than as structural, contextual, and historical experiences (e.g., cultural events; [5,26,61]. Consequently, the currently employed assessments and interventions may not adequately address the adversity and trauma experienced among racially diverse families, communities, and organizations (e.g., discrimination, institutional racism, community violence; [61]). By expanding the view of trauma, we consider the dose response relation between ACEs and other traumas and outcomes (e.g., health) among both the youth and adults. The ARCCH Model of Resilience also helps to explain the disproportionality that may be evidenced in outcomes among racial, ethnic, cultural, and linguistic minority populations. Racially and culturally responsive, trauma-informed models must be directed toward building individual, organizational, and contextual resilience and healing.

Although systems (families, communities, schools, and organizations) can induce trauma, they also can be positive and promote resilience [26,62]. Trauma and systems of oppression work in concert and thus organizations cannot be trauma-informed without a commitment to culture, equity, and social and racial justice [62]. Organizations that are resilience building carefully consider their practices, policies, and procedures. These organizations promote racial and cultural humility among their staff (e.g., school personnel) and examine how ACEs and trauma relate to organizational outcomes (e.g., school suspensions and expulsions; [63]). These systems (e.g., schools, health care) develop organizational resilience and trauma-informed, antiracist, critically race-conscious practices by recognizing how culture adds to and intersects with trauma and outcomes across levels (individual and organizational). The use of the ARCCH Model of Resilience helps organizations to be able to develop organizational resilience as described above.

#### 2.2.5. Health

As noted above, health is greatly impacted by trauma and adverse childhood experiences [1,6]. When we think of health, we have a broad understanding of it. We are thinking about the physical and mental health of the individual, family, community, and system. We also consider boundaries and what is most useful for the focus of support knowing that it can be different based on a myriad of reasons, such as cultural differences. Within health we are also thinking about what might be some additional sources of support that may need to be accessed and if referrals are needed.

#### 2.2.6. System

Within each of these components described above, we conceptualize each concept to include a systemic understanding in which any system (e.g., family, community, organization) can also benefit from these components. Attachment was broadened to sense of belonging and/or mission and vision of an organization. Regulation was broadened to understand the rules and processes within the system to help it function, and competence in the description above broadens our understanding to adults. For example, competence within a system can include what that system does well; it can also be the organization, communication, meeting the mission/vision/purpose of the system. Culture goes beyond cultural identity and cultural context to include the culture of a system. Health from a systemic perspective has to do with the overall functioning of the system. With this expansion, we are asserting that systems can also benefit from the application of the ARCCH Model of Resilience to support the systems resilience.

### 2.3. Assumptions Underlying the ARCCH Model of Resilience

Below is an overview of the four leading assumptions to the use of the ARCCH Model of Resilience.

#### 2.3.1. Interconnection between ARCCH and Systems

Our first assumption has to do with interconnectedness within two areas of the model. The first is the interconnection among those being supported by the model (i.e., whomever the focus of the model is and all those that interconnect with) and the second is the interconnection of the concepts involved in the ARCCH Model of Resilience (i.e., attachment, regulation, competence, culture, and health).

To better understand what we mean by the interconnectedness among those being supported by the model (individual, family, community, systems), we want to highlight that family systems theory tenets inform this understanding. Family systems theory proports that you cannot understand an individual without understanding the family and understanding the family as an emotional unit [64]. Each member of a family is interconnected and interdependent upon the other members of the family. The experiences we have within our family system are always with us whenever we interact with those outside our family, who also have their own emotional unit that they bring with them to the interaction. When you have multiple people interacting (whether from the same family or from different families), they are not just blank slates interacting with another blank slate, rather they are interconnected with their family and family experiences, and their experiences interact with the experiences that each other person is bringing with them. This understanding of interconnectedness is evident within each level of the model, including individual, family, community, and system. Depending upon the focus of support, understanding this level of interconnectedness is important to address and to understand how it also intersects with the interplay of one’s culture as discussed above.

To better understand the interconnectedness of the concepts of the ARCCH Model of Resilience, we also must make clear another assumption that we discussed in the strengths-based paragraph. The use and application of the ARCCH Model of Resilience is not linear. Consequently, when looking at Figure 1 and the concepts of ARCCH- attachment, regulation, competence, culture, and health, there is not one specific way in which you can start to use the model. For instance, most people will want to start with ‘attachment’ of the ARCCH Model. As humans are relational beings, this is understandable and would be a good choice in many situations. However, for those who have experienced trauma, attachment or relational connection may seem too risky and difficult to address immediately. Instead, it may be best to start support by looking at other areas of the ARCCH Model of Resilience that may have an opening with that person, either addressing their regulation, competence, culture, or health. It could be that if someone decided to start with culture, the person facilitating the model can broach [58,59,60] culture with those they are supporting. By broaching and inviting conversations about one’s culture, the person supporting the individual, family, community, or system can demonstrate their interest and respect for who they are and their experiences. From here, the person can then go to any of the concepts in ARCCH to continue to find ways to support whoever the focus of the model. Within each of the ARCCH concepts, it is important to first address what the areas of strength are. In instances where an individual, family, community, or system cannot identify a strength in that concept, it is okay to skip it for now. Over time and continued conversations, more strengths can be highlighted. It may also be that if there is a hard time identifying the strength, this is an area that needs more support. Working collaboratively with whomever is the focus of support to identify small attainable steps to help to support that area will be helpful. The goal would be to identify small attainable steps in each of the ARCCH concepts overtime to be able to fully support an individual, family, community, or system. By performing this in any one of the ARCCH concepts, you could lead conversations about the other concepts and how to support them in that area to build resilience.

#### 2.3.2. Strengths-Based Model

As stated previously this is a strength-based model that is meant to empower the individual, family, community, system that is the focus of the support. Embedded within the definition of resilience is the ability to overcome adversity [14]. The adversity and pathways to overcome adversity differ by the interplay of risk and protective factors [65]). Consequently, our model is not intended to be approached in a linear fashion. Rather, all of the ARCCH components are circular and interconnected as evidenced by the double-sided arrows in Figure 1. Depending upon the individual, family, system, or community, you can start at any of the ARCCH concepts to promote resilience. Each individual, family, community, or system has strengths or attributes that are going well that need to be acknowledged. Often trauma can be very stigmatizing, shaming, and consequently isolating [66]. Identifying and naming strengths may be difficult for some at first, but through relationships and uncovering strengths, even if it starts in just one area of the ARCCH components, can help to facilitate continued growth and identification of strengths in other components of ARCCH.

#### 2.3.3. The ARCCH Model of Resilience Is a Flexible Model

Our third assumption is that the ARCCH Model of Resilience is a flexible model. The model is flexible in that, as stated earlier, we believe that each individual, family, community, and system have their own strengths and areas for growth within the ARCCH components. While an individual’s understanding of attachment may be much more relational and a system may be much more about people’s sense of belonging, the idea of connection cuts across both. There is not one way to use this model and the application of it may change overtime even with the same individual, family, community, or system. The strengths identified or needed areas of support may change as the individual, family, community, or system grow and develop or as situations change. As each concept in the model is broad, it can hold the myriad of experiences that are identified and supported in the model.

#### 2.3.4. Multisystemic View

Lastly, the science of resilience has evolved to encompass a multisystemic view and has needed a common language and integrative framework that can be used across domains and disciplines [67]. We believe the components of ARCCH fit within any domain to increase the capabilities of multiple systems to collaborate and build resilience together. Taking the focus of resilience-building off individuals and moving it to all domains of the systems in which they are embedded is culturally responsive and congruent with the latest research in resilience, the work of happens at both an individual and systems level. Within this model, we believe that systems/organizations can utilize the ARCCH Model of Resilience to work interdisciplinarity across systems/organizations to change policies, functioning, and more holistically support the individuals, families, and communities they serve, thus leading to more cultural sensitivity and anti-bias practices, and trauma-informed practices.

### 2.4. How to Use the ARCCH Model of Resilience

As alluded to in the assumptions, the ARCCH Model of Resilience is designed to be used in a conversational, collaborative manner. It requires that the person using it is culturally responsive, trauma-informed, and committed to building resilience in either or all individuals, families, communities, and systems. Whoever is initiating the conversation and promoting resilience can use the ARCCH Model of Resilience as a guide to inform their steps. Please see Table 1 for a detailed description of the 5 steps to help use the ARCCH Model of Resilience. Step 1 is to be mindful of the setting and cultural context within which the ARCCH Model of Resilience is to be applied. What cultural considerations that need to be learned or honored first, or cultural tailoring that needs to be addressed when engaging with the individual, family, community, or system? It is important to be mindful and intentional about engaging in and being culturally responsive in our interactions with those we wish to support. Step 2 is to identify who is the focus of the ARCCH Model of Resilience; is it the individual, family, community, or system? In Step 3, you want to assess/identify the individual, family, community, or system’s strengths. As stated in the assumptions, identifying strengths may be difficult for some to engage in, particularly depending upon the type(s) of trauma they may have experienced and their current context. Therefore, it is important to validate the difficulty they may be having while trying to identify strengths, and help them to think of one, even if it is the smallest thing that they do not think is worth mentioning. Identifying just one can be helpful to help support a strength-based assessment. However, you also do not want this to be a place where someone feels invalidated if they cannot name a strength or even the smallest one. Instead, move on to Step 4 and identify what are the areas that need support. Questions can be asked about which of the ARCCH components are areas that you as an individual, family, community, or system thinks is necessary to be addressed to promote resilience. It is okay if they state that all areas need support. Within this conversation, talk with whomever is the focus and discuss specific areas in which they would be grateful for support. Talk with them to describe how the support could materialize. Lastly, Step 5, once the strengths and areas of support have been identified, then you can work collaboratively with whomever is the focus to determine small, manageable steps to build support in each area of the ARCCH Model. You will work to say, “I will work on ‘blank’ to build attachment with my parent-neighbors-colleagues” (to get at attachment) or ‘I will work to incorporate ‘blank’ into my routine so that I feel healthier” (to get at health). Two worksheets are provided in Appendix A and Appendix B to help with using the ARCCH Model of Resilience as an assessment tool. Questions can be modified based on who the focus of support is.

In the description above, it may seem that it is easier to engage in this process if the focus of support is an individual. We would argue that yes, it is more straight forward to have the conversation with an individual, however, the conversations with families, communities, and systems require a greater understanding and awareness of the assumptions we discussed about interconnectedness and what each person brings into the interaction/conversation. Awareness for and attention to the ways in which cultural tailoring may be needed depends upon who is the focus of support. Creativity may be needed to find a way to capture the voices of many different people’s experiences in a way that is authentic and validates those who are sharing their perceived strengths and areas for support. When working with different systems/organizations, we have designated different spots in the room that align with each of the ARCCH components. Then, the members of the system took markers and wrote what they thought the strengths were for each ARCCH component then for another round they listed the areas of support. The person (s) leading this then collated the information and shared it out. Then collaborative planning can occur to identify top priorities and next steps to build resilience within the system.

### 2.5. Fictional Vignette Showing Strengths and Need for Support Using the ARCCH Model

Zevin H. is a 12-year-old boy, the youngest sibling of 4 in a family who immigrated from Honduras 2 years ago. Zevin seemed to be adjusting to his school because it was a safe place where he felt that he was learning, which was important to his family. Zevin is a very curious and intelligent student. Recently, school staff have noticed Zevin’s increasing, unexcused absences, and a couple of teachers have mentioned that Zevin has been acting out physically which is uncharacteristic of him, except for his soccer coach of whom speaks favorably of him in their interactions. His mother has similar complaints, stating that Zevin is rarely on time for family meals and planned events, has started slamming doors and stomping away, and has not been doing his part to help support the family in the way that his older siblings are. The family has experienced intergenerational trauma and community violence in their home country, which has affected their sense of connectedness to each other and mistrust of those outside the family. They still struggle in transitioning to this new community, although they are loosely connected with a small, local faith community. Zevin’s behaviors are increasing, and adults at the school think that the family may be experiencing some challenging circumstances.

This vignette will be addressed through the eyes of a school team because they were the first to address Zevin’s increasing absences and increased physical behavior. Please note that schools are often the frontline for students receiving supports and services [68,69]. The team will use the ARCCH Model of Resilience as a flexible multisystemic framework to find areas of strength and what supports Zevin may need, as well as his family. The example shared below is just one way in which the ARCCH Model of Resilience might be used. The more collaborative and creative a team is that uses the ARCCH Model of Resilience the easier they will be able to come up with innumerable ways to support individuals, families, communities, and systems. Important considerations would include the developmental capacities (climate & culture) of the individual, family, community, and system as well as needs that may exist. In this example, the school team begins with a focus on the resilience shown by Zevin and his family in their survival of community violence, displacement, and navigating how they honor their cultural identity as they settle into a new culture.

#### 2.5.1. ARCCH for Individual (Zevin)

Zevin’s school employs a leadership model of teaming in which the ARCCH Model of Resilience is used to identify strengths before discussing possible supports (Appendix A). The school team agreed that Zevin has many strengths contributing to his resilience. Using the ARCCH Model of Resilience, his strengths are determined to be culture, competence, and health. For culture, his family places high value on work and a strong collectivist orientation, which is helpful to Zevin. For competence, Zevin shows curiosity and an ability to learn in the school environment and is performing at grade level academically. For health, his assessments from the physical education teachers shows Zevin to be physically healthy and developmentally on track for his age with no known health impairments. He participates in extra-curricular soccer in which he demonstrates exceptional skills (competence). Soccer is the one place he is always on time, focused, (regulation) and has not had any physical altercations with anyone. The areas that need support—although it is unclear how these areas may be related to culturally sanctioned roles and responsibilities when at home—are attachment (to other adults and to his family), regulation (to decrease physical aggression, unexcused absences, and time management), and culture (struggling with cultural identity within his own family and tensions within the new culture).

Using his strength of competence in soccer and an attachment to his soccer coach, the school team will employ the soccer coach to help build positive connections with Zevin and his family. Please note that when building resilience, it is important to consider who could be recruited to help to support the individual, family, community, or system-being as inclusive as possible. The soccer coach is also the social studies instructor in Zevin’s school, so he can use this role to build relationships with the family as well through personal invitations to school soccer games and other school events. Since Zevin displays no issues with focus or absences for soccer but also shows no signs of physical aggression in social studies class (competence), this is another area to explore how building attachment with other teachers and engagement in sports that has lots of body movement (health) may help Zevin. The coach/social studies teacher did note that his class employs frequent movement (health) and personalized, project-based learning (competence) so Zevin remains focused on the learning and follows instructions from the teacher (regulation and attachment) in this context. In other lecture-based classes, Zevin has been noted to be fidgety and unable to focus (lack of regulation). He does not respond to instructions from those teachers (lack of attachment) and sometimes refuses to stay in his seat when asked (lack of regulation), which teachers have interpreted as disrespectful, leading to further lack of attachment, although it could be that there are system-related factors (lack of culturally sustaining pedagogy) used in some of his classes and/or lack of cultural and linguistic competence of some of his teachers in his new school that related Zevin’s behaviors.

#### 2.5.2. ARCCH for the Family

The H. family consists of a mother, father, 3 teenage children, and 12-year-old Zevin. The team begins with a focus on the resilience strengths demonstrated by the H. family in all that they have overcome, leading to their desire to immigrate and discussing how they are honoring their cultural identities within a new culture. In discussing the ARCCH model with the family, the main strengths identified are competence and culture. Their culture emphasizes a strong belief in family, loyalty, and hard work, so the culture and competence are strongly related in this family. Although the father was successful in Honduras (competence), he is struggling to find full-time work, so he takes on available jobs. The mother works in a restaurant for less than minimum wage with no benefits.

After the coach/social studies teacher intentionally builds attachment through contacting the family with positive phone calls about Zevin’s success in soccer (competence), he invites them to attend games and offers free admission, which they are thankful for but do not accept. Zevin’s parents expressed that even though they know that Zevin loves soccer and thoroughly enjoyed playing throughout his childhood, they are not sure Zevin should continue soccer because the family needs him to help support the family in the way that his older siblings are doing. The school leadership team acknowledges the context with which the family is operating since they immigrated to the United States, and they also think that removing Zevin’s main source of competence and attachment in the school and connection to his culture (soccer) could be difficult for Zevin. The school leadership team uses this as an invitation to have a conversation with the family about the ARCCH Model of Resilience and ways to best support Zevin and the family. Reviewing the ARCCH model with the family helps highlight the important role that soccer has in Zevin’s attachment to school. Through the conversation they also realized that Zevin’s irrational beliefs (regulation) in being the only one unemployed has led to him distancing himself from the family, and they all determine together that this is the contributing factor to his perceived lack of attachment in the family, which also contradicts the importance of family and family relationships within his cultural identity (culture). Addressing his irrational beliefs (regulation) and rallying the family support for his sole attention on school and soccer (competence) helps to rebuild family attachment.

When looking at the ARCCH of Resilience with the family, it was clear that the family was experiencing substantial financial instability and did not have enough to pay for all the bills, so they experience food shortage and occasional loss of power. In the ARCCH Model of Resilience, we know that families do not operate in isolation. An area that needs support is attachment to the community. Given the community violence they experienced in Honduras, they have been reluctant to engage in the community in the ways in which they once would. Attachment to the community could help provide regulation and supports during this time of transition and adjustment.

#### 2.5.3. ARCCH for Community

Consistent with the ARCCH Model of Resilience, in helping the H. family to become more connected to their community, the school leadership team and the H. family had conversations about the ways in which they had been involved in the community before and what places they would prefer to get involved in their new community. Together they identified manageable and doable steps that would help them increase their civic connection in ways that were culturally sensitive and trauma informed. With this web of community support, the family was able to access temporary financial support, career counseling, energy assistance, and access to a food pantry, which increased the family’s civic competence and attachment. The family began to feel more a part of the community but also identified that they needed a faith community that aligned with their cultural beliefs. Another possible support identified by the family, school team, and community was a language barrier in need of translators. Since this rural community could not provide all the needed supports, it was necessary to reach out to a larger nearby community as well as reaching out to online communities that could meet these needs.

After using ARCCH Model of Resilience to identify the strengths in Zevin and in the family, then finding areas that need supports, health was identified as both a strength and area that needed supports from the community. Both the community health care system and the school identified a need for increased attention to regulation and culture since they were lacking in available translators and needed to make this part of their services. Implementing a translator into their health care and school helped identify previously undiagnosed attention deficit hyperactivity disorder (ADHD) that was contributing to Zevin’s increasing inability to keep focus with his growing commitments in school and family, thereby showing health to be both a strength and a need for collaborative support from both the school and health care providers.

#### 2.5.4. ARCCH for the System

To understand the ARCCH Model of Resilience for the system (Appendix B), we can think about it in two ways, (a) from a multiple systems perspective, or (b) from within any of the systems that Zevin and his family interact. First, we will talk about ARCCH Model of Resilience from a multiple systems perspective. The importance of collaboration across systems became more apparent with the way to support Zevin and his family. The greater number of systems, such as the school system, health care system, non-profits, and so forth that can utilize a common resilience framework can provide a common language to work across systems to support individuals, families, and communities. Secondly, in this scenario, while the school was not the focus of the support, the school used the ARCCH Model of Resilience to support students and families. The school’s ability to support Zevin and his family in this way occurred because of the work they have conducted to identify their own ARCCH. They knew which teacher to connect with Zevin because they monitor which students have relationships with which adults in the building. They also operate from a mindset of being a community hub and work to ensure that they have community connections to refer families to and maintain the relationships within the community to ensure that the services they recommend or refer are trustworthy and culturally responsive. The school was aware of their own regulation processes, timeliness, grades, rules surrounding soccer practice, to know what the best way to navigate supporting Zevin. The school was aware of providing a very broad understanding of competence to look at students holistically, thus noticing Zevin’s competence in soccer and area of connection for him. Culturally, the school being connected to the community was careful to be culturally responsive and to tailor the conversation with Zevin’s parents according to their experiences. Lastly, for health, they had good referral sources and were culturally responsive in partnering with Zevin’s family to advocate for greater linguistic accessibility in their community, thus resulting in medical supports for Zevin.

## 3. Conclusions

Trauma is a pervasive and prevalent concern that impacts the health and well-being of individuals, families, and systems and disproportionately impacts racial and ethnic minority populations. Strengthening and supporting the resilience of individuals, families, and systems is necessary to improve society. In this paper, we have proposed the ARCCH Model of Resilience, a culturally responsive, trauma informed, flexible framework that can be used in different settings and contexts to support resilience of individuals, families, communities, and systems. The use of this model will help guide a variety of professionals and laypeople to use a common language to have meaningful conversations to identify the individual, family, community, and system strengths, identify what areas of support are needed, and to name realistic manageable steps to promote resilience.

## Figures and Tables

**Table 1:** 5 Step Guide to Using the ARCCH Model of Resilience.

Steps	What to Assess/Ask Questions About	Information and/or Potential Questions/Prompts to Consider
Step 1	Identify the setting and cultural context that ARCCH will be applied	The facilitator should first do their own homework to gather whatever information they can prior to the first meeting. Is there something about the organization that is important to understand? Are there historical traumas that are present? One should not expect whomever is the focus of support to teach them the foundation. Instead, the facilitator’s job is to help to understand the nuances for each person(s) involved through the conversation.
Step 2	Identify who the focus of support is: the individual, family, community, or system	Depending upon how the connection was made, this may be obvious. However, it is important to consider the interconnection of individuals, families, communities, and systems. Whomever may be the focus at the start of the conversation or support may not be the sole area of focus. The flexibility to move between all involved and to see it from a systemic perspective will be valuable.
Step 3	Identify the strengths of whomever is the focus of the support	Individual Attachment: Can you tell me who you are closest to? What is it about that person that helps you feel close to them? Family Culture: I would be curious to learn from each of you what aspects of your family’s culture and background provides you with greatest sense of pride?
Step 4	Identify what areas are in need of support	System Regulation: I know that within organizations there are a lot of moving parts and often a lot of expertise about ways in which things can be improved upon. Could you each tell me a little bit about areas within your policies and procedures you would like to see improved?
Step 5	Once areas of strength and support are identified then you can collaboratively create small manageable steps to build support each area. Please see Appendix A and Appendix B to help facilitate conversation.	It is important to note that it may not seem feasible in the first or even after multiple conversations to feel like you have a solid plan of support for each area. Please know it is ok to focus on one area for the time it needs. Then once someone is feeling confident in that area, it could be possible to build off that into another area of the ARCCH components.

Please note that all these steps and the information you gather are likely to change overtime. Please use these steps as a flexible guide and reassess/reengage around conversations as it is appropriate.

**Figure A1:**
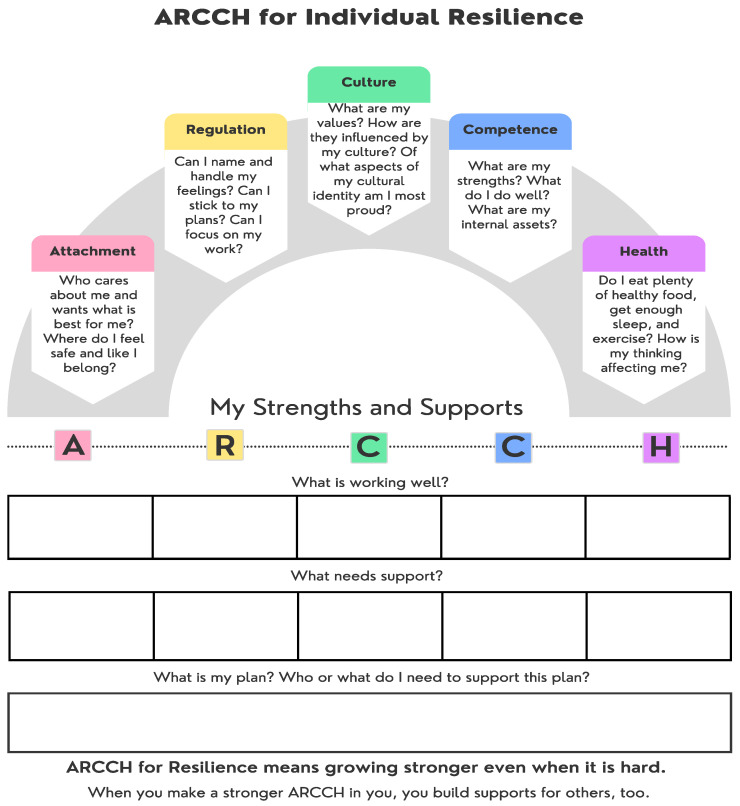
Worksheet to help guide ARCCH conversation with an individual.

**Figure A2:**
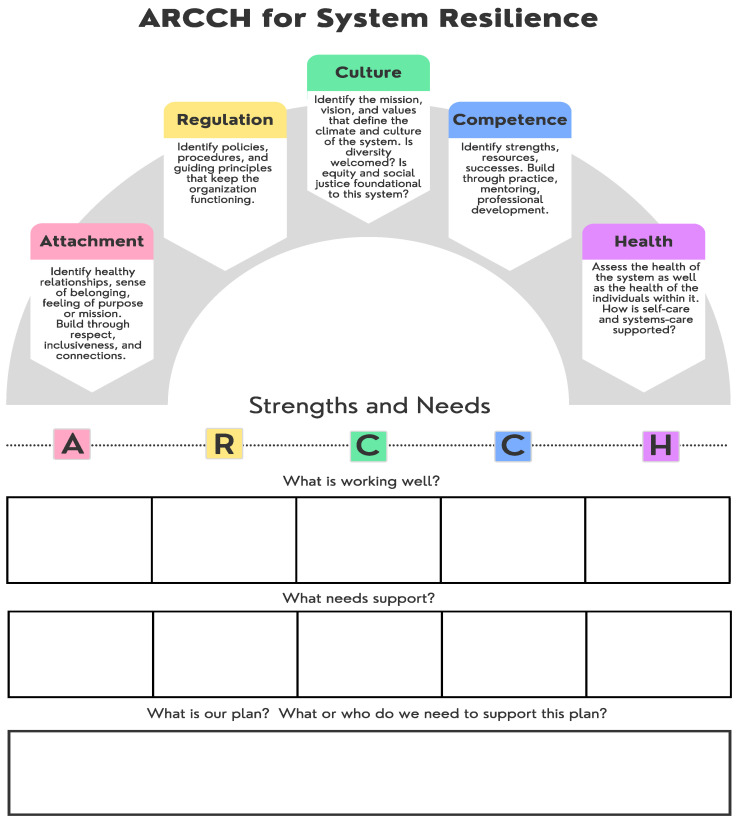
Worksheet to help guide ARCCH conversation with an organization/system.

## Data Availability

Not applicable.

## References

[1] Felitti V.J., Anda R.F., Nordenberg D., Williamson D.F., Spitz A.M., Edwards V., Koss M.P., Marks J.S. (1998). REPRINT OF: Relationship of Childhood Abuse and Household Dysfunction to Many of the Leading Causes of Death in Adults: The Adverse Childhood Experiences (ACE) Study. Am. J. Prev. Med..

[2] Substance Abuse and Mental Health Services Administration (2014). SAMHSA’s Concept of Trauma and Guidance for a Trauma-Informed Approach. HHS Publication No. (SMA) 14-4884. Rockville, MD: Author. https://ncsacw.samhsa.gov/userfiles/files/SAMHSA_Trauma.pdf.

[3] Hughes K., Bellis M.A., Hardcastle K.A., Sethi D., Butchart A., Mikton C., Jones L., Dunne M.P. (2017). The effect of multiple adverse childhood experiences on health: A systematic review and meta-analysis. Lancet Public Health.

[4] Danese A., McEwen B.S. (2012). Adverse childhood experiences, allostasis, allostatic load, and age-related disease. Physiol. Behav..

[5] Cronholm P.F., Forke C.M., Wade R., Bair-Merritt M.H., Davis M., Harkins-Schwartz M., Pachter L.M., Fein J.A. (2015). Adverse Childhood Expereince: Expanding the concept of adversity. Am. J. Prev. Med..

[6] Sacks V., Murphey D. (2018). The Prevalence of Adverse Childhood Experiences, Nationally, by State, and by Race or Ethnicity. http://hdl.handle.net/20.500.11990/1142.

[7] Centers for Disease Control and Prevention (2021). The Social-Ecological Model: A Framework for Prevention. https://www.cdc.gov/violenceprevention/about/social-ecologicalmodel.html.

[8] Bernard D.L., Calhoun C.D., Banks D.E., Halliday C.A., Hughes-Halbert C., Danielson C.K. (2021). Making the “C-ACE” for a Culturally-Informed Adverse Childhood Experiences Framework to Understand the Pervasive Mental Health Impact of Racism on Black Youth. J. Child Adolesc. Trauma.

[9] Ungar M., Theron L., Murphy K., Jefferies P. (2021). Researching Multisystemic Resilience: A Sample Methodology. Front. Psychol..

[10] Baker F.R.L., Baker K.L., Burrell J. (2021). Introducing the skills-based model of personal resilience: Drawing on content and process factors to build resilience in the workplace. J. Occup. Organ. Psychol..

[11] Maurović I., Liebenberg L., Ferić M. (2020). A review of family resilience: Understanding the concept and operationaliza-tion challenges to inform research and practice. Child Care Pract..

[12] Ellis W.R., Dietz W.H. (2017). A New Framework for Addressing Adverse Childhood and Community Experiences: The Building Community Resilience Model. Acad. Pediatr..

[13] Milken Institute School of Public Health (2017). Building Community Resilience: Coalition Building and Communications Guide. https://publichealth.gwu.edu/sites/default/files/downloads/Redstone-Center/BCR%20Coalition%20Building%20and%20Communications%20Guide.pdf.

[14] Masten A.S., Lucke C.M., Nelson K.M., Stallworthy I.C. (2021). Resilience in development and psychopathology: Multisystem perspectives. Annu. Rev. Clin. Psychol..

[15] Fitzgerald H.E., Johnson D.J., Allen J., Villarruel F.A., Qin D.B. (2021). Historical and race-based trauma: Rsilience through family and community. Advers. Resil. Sci..

[16] Kinniburgh K., Blaustein M. (2005). Attachment, self-regulation, and competence: A comprehensive framework for intervention with complexly traumatized youth. A treatment manual. Psychiatr. Ann..

[17] Kinniburgh K.J., Blaustein M., Spinazzola J., van der Kolk B.A. (2005). Attachment, Self-Regulation, and Competency: A comprehensive intervention framework for children with complex trauma. Psychiatr. Ann..

[18] Blaustein M., Kinniburgh K. (2010). Treating Traumatic Stress in Children and Adolescents: How to Foster Resilience through Attachment, Self-Regulation, and Competence.

[19] Ford J.D., Blaustein M.E. (2013). Systemic Self-Regulation: A Framework for Trauma-Informed Services in Residential Juvenile Justice Programs. J. Fam. Violence.

[20] Brend D., Fréchette N., Milord-Nadon A., Harbinson T., Collin-Vezina D. (2020). Implementing trauma-informed care through social innovation in residential care facilities serving elementary school children. Int. J. Child Adolesc. Resil..

[21] Hodgdon H.B., Kinniburgh K., Gabowitz D., Blaustein M.E., Spinazzola J. (2013). Development and Implementation of Trauma-Informed Programming in Youth Residential Treatment Centers Using the ARC Framework. J. Fam. Violence.

[22] Hodgdon H.B., Blaustein M., Kinniburgh K., Peterson M.L., Spinazzola J. (2016). Application of the ARC Model with Adopted Children: Supporting Resiliency and Family Well Being. J. Child Adolesc. Trauma.

[23] Dorado J.S., Martinez M., McArthur L.E., Leibovitz T. (2016). Healthy Environments and Response to Trauma in Schools (HEARTS): A Whole-School, Multi-level, Prevention and Intervention Program for Creating Trauma-Informed, Safe and Supportive Schools. Sch. Ment. Health.

[24] Arvidson J., Kinniburgh K., Howard K., Spinazzola J., Strothers H., Evans M., Andres B., Cohen C., Blaustein M.E. (2011). Treatment of Complex Trauma in Young Children: Developmental and Cultural Considerations in Application of the ARC Intervention Model. J. Child Adolesc. Trauma.

[25] Hampton-Anderson J.N., Carter S., Fani N., Gillespie C.F., Henry T.L., Holmes E., Lamis D.A., LoParo D., Maples-Keller J.L., Powers A. (2021). Adverse childhood experiences in African Americans: Framework, practice, and policy. Am. Psychol..

[26] Saleem F.T., Howard T.C., Langley A.K. (2021). Understanding and addressing racial stress and trauma in schools: A pathway toward resistance and healing. Psychol. Sch..

[27] American Psychological Association, APA Task Force on Race and Ethnicity Guidelines in Psychology (2019). Race and Ethnicity Guidelines in Psychology: Promoting Responsiveness and Equity. https://www.apa.org/about/policy/race-and-ethnicity-in-psychology.pdf.

[28] Henfield M., Washington A.R., Besirevic Z., De La Rue L. (2019). Introduction to Trauma-Informed Practices for Mental Health and Wellness in Urban Schools and Communities. Urban Rev..

[29] Scrine E. (2021). The Limits of Resilience and the Need for Resistance: Articulating the Role of Music Therapy with Young People within a Shifting Trauma Paradigm. Front. Psychol..

[30] Shonkoff J.P., Slopen N., Williams D.R. (2021). Early Childhood Adversity, Toxic Stress, and the Impacts of Racism on the Foundations of Health. Annu. Rev. Public Health.

[31] Petrone R., Stanton C.R. (2021). From producing to reducing trauma: A call for “trauma-informed” research (ers) to in-terrogate how schools harm students. Educ. Res..

[32] Centers for Disease Control and Prevention About the CDC-Kaiser ACE Study. https://www.cdc.gov/violenceprevention/childabuseandneglect/acestudy/about.html.

[33] Johnson S.R. Kaiser Pledges $2.75 Million to Research Childhood Trauma. https://www.modernhealthcare.com/providers/kaiser-pledges-275-million-research-childhood-trauma.

[34] Metzler M., Merrick M.T., Klevens J., Ports K.A., Ford D.C. (2017). Adverse childhood experiences and life opportunities: Shifting the narrative. Child. Youth Serv. Rev..

[35] Merrick J.S., Narayan A.J., DePasquale C.E., Masten A.S. (2019). Benevolent Childhood Experiences (BCEs) in homeless parents: A validation and replication study. J. Fam. Psycho..

[36] Ungar M. (2021). Modeling multisystemic resilience: Connecting biological, psychological, social, and ecological adaptation in contexts of adversity. Multisystemic Resilience: Adaptation and Transformation in Contexts of Change.

[37] Shonkoff J.P. (2016). Capitalizing on Advances in Science to Reduce the Health Consequences of Early Childhood Adversity. JAMA Pediatr..

[38] Thomason M.E., Marusak H.A. (2017). Toward understanding the impact of trauma on the early developing human brain. Neuroscience.

[39] Banyard V., Hamby S., Grych J. (2017). Health effects of adverse childhood events: Identifying promising protective factors at the intersection of mental and physical well-being. Child Abus. Negl..

[40] Portilla X.A., Ballard P.J., Adler N.E., Boyce W.T., Obradović J. (2014). An Integrative View of School Functioning: Transactions between Self-Regulation, School Engagement, and Teacher-Child Relationship Quality. Child Dev..

[41] Berástegui A., Pitillas C., Ungar M. (2021). What does it take for early relationships to remain secure in the face of adversity? At-tachment as a unit of resilience. Multisystemic Resilience: Adaptation and Transformation in Contexts of Change.

[42] Xia M., Fosco G.M., Feinberg M.E. (2016). Examining reciprocal influences among family climate, school attachment, and academic self-regulation: Implications for school success. J. Fam. Psychol..

[43] Brown S.M., Baker C.N., Wilcox P. (2012). Risking connection trauma training: A pathway toward trauma-informed care in child congregate care settings. Psychol. Trauma Theory Res. Pract. Policy.

[44] Townley C. (2021). Inclusion, belonging and intercultural spaces: A narrative policy analysis of playgroups in Australia. Aust. J. Soc. Issues.

[45] Escalera-Reyes J. (2020). Place Attachment, Feeling of Belonging and Collective Identity in Socio-Ecological Systems: Study Case of Pegalajar (Andalusia-Spain). Sustainability.

[46] Anyon Y., Atteberry-Ash B., Yang J., Pauline M., Wiley K., Cash D., Downing B., Greer E., Pisciotta L. (2018). It’s All about the Relationships”: Educators’ Rationales and Strategies for Building Connections with Students to Prevent Exclusionary School Discipline Outcomes. Child. Sch..

[47] Theron L., Ungar M. (2021). Learning about systemic resilience from studies of student resilience. Multisystemic Resilience: Adaptation and Transformation in Contexts of Change.

[48] Zelazo P.D., Blair C.B., Willoughby M.T. (2016). Executive Function: Implications for Education (NCER 2017–2000).

[49] Hambrick E.P., Brawner T.W., Perry B.D., Brandt K., Hofmeister C., Collins J.O. (2019). Beyond the ACE score: Examining relationships between timing of developmental adversity, relational health and developmental outcomes in children. Arch. Psychiatr. Nurs..

[50] Anda R.F., Fleisher V.I., Felitti V.J., Edwards V.J., Whitfield C.L., Dube S.R., Williamson D.F. (2004). Childhood abuse, household dysfunction, and indicators of impaired adult worker performance. Perm. J..

[51] Brunzell T., Stokes H., Waters L. (2016). Trauma-Informed Positive Education: Using Positive Psychology to Strengthen Vulnerable Students. Contemp. Sch. Psychol..

[52] Blitz L., Yull D., Clauhs M. (2016). Bringing Sanctuary to School: Assessing School Climate as a Foundation for Culturally Responsive Trauma-Informed Approaches for Urban Schools. Urban Educ..

[53] Bloom S.L. (1997). Creating Sanctuary: Toward the Evolution of Sane Societies.

[54] Wojciak A.S., Powers J., Medberry L., Reardon M., Leonard J. (2020). Schoolwide trauma informed professional development: We Can! Building Relationships and Resilience. Alleviating the Educational Impact of Adverse Childhood Experiences: School-University-Community Collaboration.

[55] Howse R.B., Lange G., Farran D.C., Boyles C.D. (2003). Motivation and Self-Regulation as Predictors of Achievement in Economically Disadvantaged Young Children. J. Exp. Educ..

[56] Taxer J.L., Gross J.J. (2018). Emotion regulation in teachers: The “why” and “how”. Teach. Teach. Educ..

[57] Van der Heijde C.M. (2014). Employability and Self-Regulation in Contemporary Careers. Psycho-Social Career Meta-Capacities.

[58] Day-Vines N.L., Cluxton-Keller F., Agorsor C., Gubar A. (2021). Strategise for Broaching the subjects of race, ethnicity, and culture. J. Couns. Dev..

[59] Day-Vines N.L., Wood S.M., Grothaus T., Craigen L., Holman A., Dotson-Blake K., Douglass M.J. (2007). Broaching the Subjects of Race, Ethnicity, and Culture During the Counseling Process. J. Couns. Dev..

[60] Bryant C.M., Anderson L.A., Notice M.R. (2022). Revisioning the Concept of Resilience: Its Manifestation and Impact on Black Americans. Int. J. Fam. Ther..

[61] Wade R., Shea J.A., Rubin D., Wood J., Coker T.R., Moreno C., Shekelle P.G., Schuster M.A., Chung P.J. (2014). Adverse Childhood Experiences of Low-Income Urban Youth. Pediatrics.

[62] Altman L. (2021). Building an organization that promotes healing, well-being, and resilience. J. Public Health Manag. Pract..

[63] Pierce H., Jones M.S., Gibbs B.G. (2022). Early adverse childhood experiences and exclusionary discipline in high school. Soc. Sci. Res..

[64] Bowen M. (1978). Family Therapy in Clinical Practice.

[65] Cicchetti D. (2010). Resilience under conditions of extreme stress: A multilevel perspective. World Psychiatry.

[66] Schomerus G., Schindler S., Rechenberg T., Gfesser T., Grabe H.J., Liebergesell M., Sander C., Ulke C., Speerforck S. (2021). Stigma as a barrier to addressing childhood trauma in conversation with trauma survivors: A study in the general population. PLoS ONE.

[67] Masten A.S. (2016). Resilience in developing systems: The promise of integrated approaches. Eur. J. Dev. Psychol..

[68] Gott J., Lang P. (2003). The school: The front line of mental health development. Pastor. Care Educ..

[69] Rothì D.M., Leavey G., Best R. (2008). On the front-line: Teachers as active observers of pupils’ mental health. Teach. Teach. Educ..

